# Theoretical distinction between functional states in working memory and their corresponding neural states

**DOI:** 10.1080/13506285.2020.1825141

**Published:** 2020-09-24

**Authors:** Mark G. Stokes, Paul S. Muhle-Karbe, Nicholas E. Myers

**Affiliations:** Wellcome Centre for Integrative Neuroimaging and Department of Experimental Psychology, University of Oxford, Oxford, UK

**Keywords:** Working memory, latent information storage, activity-silent

## Abstract

Working memory (WM) is important for guiding behaviour, but not always for the next possible action. Here we define a WM item that is currently relevant for guiding behaviour as the functionally “active” item; whereas items maintained in WM, but not immediately relevant to behaviour, are defined as functionally “latent”. Traditional neurophysiological theories of WM proposed that content is maintained via persistent neural activity (e.g., stable attractors); however, more recent theories have highlighted the potential role for “activity-silent” mechanisms (e.g., short-term synaptic plasticity). Given these somewhat parallel dichotomies, functionally active and latent cognitive states of WM have been associated with storage based on persistent-activity and activity-silent neural mechanisms, respectively. However, in this article we caution against a one-to-one correspondence between functional and activity states. We argue that the principal theoretical requirement for active and latent WM is that the corresponding neural states play qualitatively different functional roles. We consider a number of candidate solutions, and conclude that the neurophysiological mechanisms for functionally active and latent WM items are theoretically independent of the distinction between persistent activity-based and activity-silent forms of WM storage.

## Introduction

Information held in working memory (WM) is usually relevant for guiding future behaviour, but not necessarily the next upcoming action. Consider the following scenario: After giving a talk, an audience member asks you a two-part question. As you are preparing to answer question 1, you need to avoid distraction from question 2, lest it interfere with your first answer. However, question 2 still needs to be stored in memory so that you can answer it eventually. Ultimately, both items are of equal importance. Both need to be robustly encoded and maintained, but only the relevant one should directly influence your current behaviour. Here we define the memory item that is currently relevant as the “active” item, in the sense that it actively guides ongoing behaviour. By contrast, we define items maintained but not acted upon as “latent” items, meaning that they should not influence current processing. Latent items have the potential to be brought into an active state once the need arises, but until then are stored in a robust yet dormant format.^1^

Until recently, dominant neural models of WM required some neural activity to persist throughout the retention interval to maintain mnemonic information – with the possible exception of temporary gaps in activity that could be bridged by very short-lived phenomena, such as refractory periods (Amit & Brunel, 1997; Camperi & Wang, 1998; Wang, 2001; Wimmer et al., 2014). More recently, theorists have proposed that WM could also be maintained via “activity-silent” neural states, such as short-term synaptic plasticity (Bouchacourt & Buschman, 2019; Manohar et al., 2019; Zucker & Regehr, 2002). Although these models are not necessarily mutually exclusive, the apparent dichotomy between persistent activity and activity-silent mechanisms clearly resembles the functional distinction between active and latent cognitive states of WM (see next section). The purpose of this article is to caution against a direct correspondence between these functional and neural distinctions. We argue that the principal requirement for distinguishing functionally active from latent WM is that the neural state of active WM should engage with ongoing processing, whereas latent WM should be functionally inert, i.e., not interfere with ongoing processes. Here we consider potential neural solutions for this and outline how they could be tested empirically.

### Various neural mechanisms can support functionally latent working memory

The distinction between persistently active and activity-silent mechanisms of WM storage has generated vigorous debate (Constantinidis et al., 2018; Lundqvist et al., 2018), leading some to propose that the two neural mechanisms could serve distinct functions in the service of WM (Masse et al., 2019; Trübutschek et al., 2019). One popular proposal has been that persistent activity could be associated with attended items in WM, while other items are maintained in a more passive, activity-silent state (Kamiński & Rutishauser, 2019; LaRocque et al., 2013; LaRocque et al., 2015; Lewis-Peacock et al., 2012; Manohar et al., 2019; Olivers et al., 2011; Stokes, 2015). Although this view has intuitive appeal, the logic that functional WM states should align with this particular dichotomy of activity states has not been thoroughly evaluated in the literature.

In order to fulfil our operational definition, an active WM item should be readily available to interact with ongoing processing, whereas the latent WM item should have minimal influence. Critically, both active and latent items need to be maintained robustly. Further, latent items need to be available to be reformatted into an active state that allows them to affect behaviour when needed.

### Functionally active and latent states differentially engage with ongoing processing

Behavioural studies are the main source of evidence that processing of subsequent stimuli is more reliably influenced by active WM than by latent WM. Many of these studies are based on the observation that WM can maintain templates that guide attention to memory-matching stimuli (Dowd et al., 2017; Downing, 2000; Olivers et al., 2006; Soto et al., 2005; Soto et al., 2008). This effect seems to require maintenance of WM items in an active state (Olivers et al., 2011; Van der Stigchel & Olivers, 2019), since the attention-guiding effect rapidly subsides over multiple trial repetitions or stimulus presentations (Gayet et al., 2017; Kang & Spitzer, 2020; van Moorselaar, Theeuwes, & Olivers, 2016), presumably as the active WM item is reformatted into a non-interfering representation.

When multiple items are concurrently held in WM at different priority levels, only the active WM item appears to guide attention, while latent WM has only temporary influence (Mallett & Lewis-Peacock, 2018) or no influence at all (Greene et al., 2015; van Loon et al., 2017; van Moorselaar et al., 2014). Nevertheless, some studies have shown that multiple WM items can simultaneously guide attention (Hollingworth & Beck, 2016; Carlisle & Woodman, 2019), leaving open the possibility that, in some circumstances, latent WM items may have an unintended effect on cognition or behaviour (possibly as an effect of imperfect separation between active and latent WM via non-orthogonal coding schemes, see below).

A second line of convergent behavioural evidence comes from studies showing that novel task sets held in WM can automatically influence processing of subsequent stimuli in a secondary task performed in the maintenance delay (Liefooghe et al., 2012; Meiran et al., 2012; Muhle-Karbe et al., 2016). This effect seems to be specific to task sets held in active WM after they have been cued as relevant, while uncued task sets have no such effect (González-García et al., 2020).

By contrast, less is known about the neural basis of differential effects of active and latent WM on stimulus processing. In general, stimuli matching the contents of WM elicit larger neural responses (Awh et al., 2000; Gayet et al., 2017), consistent with increased deployment of attention. Similarly, maintaining a task set leads to motor-preparatory responses (lateralized readiness potentials) when the instructed stimuli are encountered in a secondary task, signalling that they are being processed according to the maintained stimulus-response mapping (Meiran et al., 2015), again suggesting that WM maintenance influences processing of subsequent stimuli.

At present, it remains unclear how specific these neural effects are to active WM because few studies have compared active and latent WM. A study manipulating the task-relevance of WM items found that neural markers of attention were amplified towards stimuli matching a WM item when it was task-relevant (Carlisle & Woodman, 2013), possibly because the item was maintained in active WM. In a recent EEG study, we aimed to measure more directly how active and latent WMs influence WM-guided behaviour (Muhle-Karbe et al., 2020). Participants held two items in WM and were cued on each trial which item was active and should be compared to a probe stimulus, while the other item was latent and maintained merely for later use. Both active and latent WM items could be decoded from patterns of EEG activity, but only the quality of representation of the active item directly affected behaviour. When an item was in the active state, trial-to-trial variability in decoding predicted the efficiency of WM-based decision-making: stronger decoding of the active item led to faster performance on that trial. This was not the case for latent items, where variability in decoding did not predict behaviour on the current trial. Interestingly, however, decoding did reflect how well the item would be remembered on other trials when it was in an active state. This distinction is consistent with the idea that active and latent forms of WM storage have distinct effects on behaviour, and that latent WM minimally interferes with ongoing processing, in line with the behavioural studies cited above.

**Figure 1.**
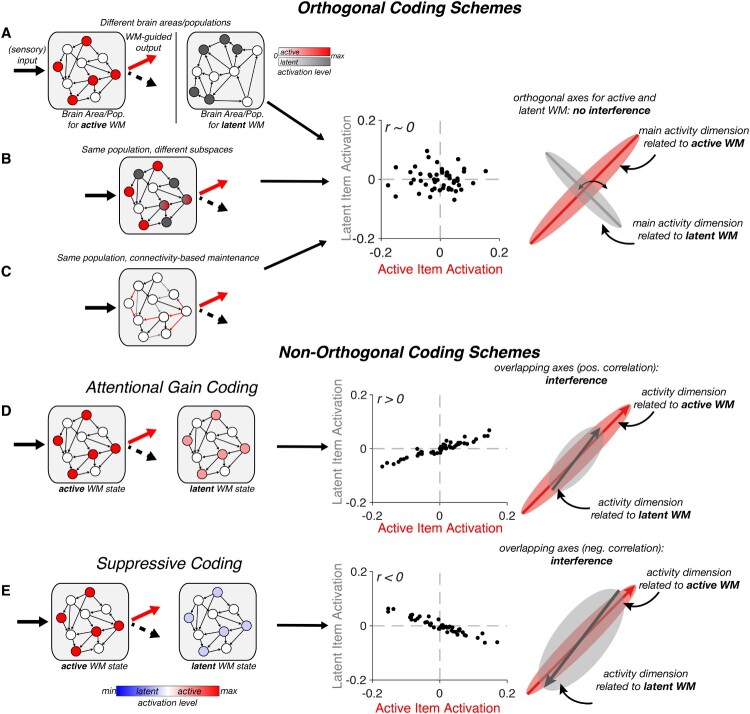
Summary of possible coding schemes for active vs. latent WM. Rows show different putative coding mechanisms for active versus latent WM. Left-hand column: Circuit-level depiction of various coding schemes in an example neural population. Each grey square represents a WM-coding neural population. Within the population, circles represent coding units (neurons), and arrows represent directed connections. Activated units are shown in colour (active: red, latent: grey or blue). Middle column: Correlation between activation patterns for items in an active (x-axis) or a latent (y-axis) state. Individual points indicate units. Correlations are exaggerated for illustration. Right-hand column: Neural state-space representation. When reduced to their most informative dimensions, neural patterns for active or latent items may occupy different subspaces. The extent of their overlap is a reflection of how correlated patterns are for active and latent WM. (A-C). Various coding schemes leading to orthogonal representations (no correlation between active and latent patterns). (A). Separate brain areas or separable neural populations. (B). Separate patterns in the same neural population. (C). Connectivity-based (i.e., activity-silent storage) can also separate active from latent WM by changing the weights of different connections in the population. (D-E). Non-orthogonal coding schemes. (D). Attention Gain coding separates active from latent WM through differences in amplitude, rather than different patterns. (E) Similarly, suppressive coding stores latent WM in the same neural pattern, but through a reversal of activity, leading to anti-correlated activity patterns that nevertheless occupy the same neural subspace.

Although this study was focused on WM-based decision dynamics rather than on maintenance per se, it does highlight the key functional distinction between active and latent WM states. Below we discuss a number of neural mechanisms that could give rise to the maintenance of WM items for such functionally distinct cognitive states. First, we discuss mechanisms that segregate active and latent items in discrete neural patterns, from separation at the large-scale anatomical level, to separation of activity subspaces within the same neural population, to the frequently invoked separation via neurophysiological mechanism: persistent activity vs activity-silent states. The key property of all three mechanisms is that active and latent WM states are statistically uncorrelated (i.e., orthogonal, see also Figure 1), ensuring that active WM states can drive behaviour independently of latent WM states. Next, we discuss non-independent (i.e., non-orthogonal) coding schemes that have been proposed recently, where there is a statistical dependence between active and latent WM states, and how these might have implications for interference from latent WM on behaviour. Finally, we briefly discuss how an alternate framework, based on separating WM representations in different phases of neural oscillations, might address the issue.

## Orthogonal coding schemes for active and latent WM

If active and latent WM have different functional properties, then by definition there must be a difference in the respective neural representation. More specifically, if an item is active in one context, but latent in another context, there must be a distinct coding scheme for the same information in each context (active vs latent; see Figure 1). If they were represented in exactly the same neural state, they would have exactly the same functional properties. Further, we suggest that the difference between active and latent WM states should be *qualitative*, not just *quantitative*. Both active and latent memories need to be maintained robustly: the key difference in not the strength of the coding, but its functional properties. Finally, if active and latent neural states are maintained by independent, or orthogonal, coding schemes, interference should be minimized.

### Anatomical separation

Perhaps the simplest means by which the brain could maintain active and latent WM items independently is via storage in distinct brain areas (Figure 1A, left). Consistent with this idea, a recent fMRI study found that active items could be decoded from BOLD activity patterns in a distributed network comprising early visual, parietal, and prefrontal areas. By contrast, latent items could be decoded from activity patterns in the intraparietal sulcus and the frontal eye fields (Christophel et al., 2018), suggestive of a division of labour between brain areas in coding for active and latent WM items. In principle, these findings meet our criterion for qualitatively different coding schemes (Figure 1A, middle and right), since behaviour could be selectively driven by those brain areas representing only the active item. Testing how decoding strength in different brain areas relates to the quality of WM-based decisions will be central to evaluating the anatomical separation hypothesis. Ultimately, causal interventions (e.g., Daie et al., 2019) may help determine whether only regions exclusively coding for active WM drive behaviour.

It is also important to note that fMRI findings of decodable delay signals do not strictly imply that WM items are encoded in persistent activity, rather than in “activity silent” states. Both could in principle result in decodable patterns in the BOLD response: Persistent activity should drive statistically separable patterns across voxels, but it is also possible that activity-silent states can be detected in separable patterns of spontaneous activity (Sugase-Miyamoto et al., 2008). Given the indirect coupling of spiking activity and BOLD (e.g., Logothetis et al., 2001), there are probably even more indirect possibilities that complicate the correspondence between decodable BOLD signals and the underlying neurophysiological mechanism. Nevertheless, whatever the underlying activity state, maintenance in anatomically segregated brain areas fulfils our basic orthogonality requirement.

### Separation by different subpopulations (or subspaces) within brain areas

A second possibility is that active and latent WM items are stored within the same brain area(s), or even in an overlapping neural population, without causing interference (Figure 1B). The main prerequisite for this coding scheme is that item-specific activity patterns for the active item are uncorrelated with item-specific patterns when the same item is latent (i.e., active and latent states are statistically separable). This may correspond to latent item patterns occupying the null space of the optimal readout weights for the active item. While the theoretical appeal of such a coding scheme has been noted (Druckmann & Chklovskii, 2012; Spaak et al., 2017), supporting evidence is still relatively scarce, and human evidence is still lacking. It is worth noting that such a scheme would imply that some cells in the population exhibit nonlinear mixed selectivity (Fusi et al., 2016) since their response to a particular WM item would depend on its functional state. The involvement of mixed selectivity in WM maintenance has been demonstrated in other contexts (Parthasarathy et al., 2017; Rigotti et al., 2013), where mixed selectivity neurons have been shown to be of particular relevance to behaviour, highlighting the potential utility of context-dependent codes.

A recent WM study recording neurons in prefrontal, parietal, and visual cortex (Panichello & Buschman, 2020) found that cueing a WM item as relevant led to a transformation of activity into a new subspace that was orthogonal to the subspace coding for the same item prior to the cue. This result can be interpreted as evidence that orthogonalisation plays a role in distinguishing active from latent WM. In a related study (Yoo & Hayden, 2020) recording from neurons in the orbitofrontal and ventromedial prefrontal cortex, two stimuli that were both needed for a reward-guided decision were maintained across a delay period in orthogonal subspaces. This separation within the same neural populations could allow downstream brain areas to be driven entirely by one stimulus without interference from the other. A similar mechanism has been demonstrated in movement planning, where premotor cortex maintains a planned movement in a latent state that is “invisible” to the motor cortex until it needs to be executed (Elsayed et al., 2016; Kaufman et al., 2014). Notably, as for the anatomical coding scheme outlined above, such orthogonal patterns could be maintained both via persistent activity, or through activity-independent means (e.g., Hopfield, 1982).

### Separation by neurophysiological mechanism

Finally, we consider the proposal that functionally active states are supported by elevated neural activity, whereas functionally latent states correspond to activity-silent mnemonic mechanisms. This distinction has been suggested by a number of authors (e.g., Kamiński & Rutishauser, 2019; LaRocque et al., 2013, 2015; Lewis-Peacock et al., 2012; Manohar et al., 2019; Olivers et al., 2011; Stokes, 2015). In the framework developed here, this division of labour is only helpful if it confers differential functional properties on active and latent WMs. As highlighted above, it does not bear on the basic maintenance demands: both active and latent memories need to be maintained robustly. Nevertheless, a division of labour between different candidate neurophysiological mechanisms (a stable attractor state based on persistent activity, or short-term synaptic plasticity) could satisfy our key requirement for orthogonal representation (Figure 1C). However, it is also often further assumed that the neurophysiological dichotomy between persistent activity and activity-silent maintenance naturally aligns with the functional dichotomy between active and latent WM. The intuition seems to be that elevated neural activity uniquely influences WM-guided behaviour (e.g., via changes in state-dependent processing), and therefore is better suited to active WM, whereas activity-silent mechanisms are effectively functionally dormant (see, e.g., Lewis-Peacock et al., 2012). However, it is important to point out that activity-silent mechanisms are not inherently functionally dormant. On the contrary, temporary changes in synaptic connectivity can have a direct functional impact on subsequent processing. For instance, encoding an active item via altered responsivity in the relevant network could allow subsequent input (i.e., a memory probe) to evoke activity that will generate an appropriate response (Stokes et al., 2013), without requiring sustained activity (Manohar et al., 2019; Mongillo et al., 2008). Therefore, both forms of maintenance (persistent activity, e.g., Mante et al., 2013; Remington et al., 2018, and activity-silent connectivity patterns, e.g., Bouchacourt & Buschman, 2019; Manohar et al., 2019) can guide decision-making and support active WM states. Similarly, as we have described above, functionally latent representations could also be maintained via persistent activity as long as they are qualitatively distinct from the corresponding active representation.

It is also important to note that other factors likely determine the extent to which a WM item is associated with persistent activity or activity-silent states. For example, it has recently been proposed that elevated activity could be a signature of current processing or transformation of WM items, rather than storage per se (Masse et al., 2020). If such transformations are more likely to occur on active WM items (as has been proposed previously, see Lewis-Peacock et al., 2012), this could explain common findings such as active WM being decoded from BOLD activity, while latent items are not.

## Non-orthogonal coding schemes

A number of alternative proposals for neural differences between active and latent items fall into the general category of non-orthogonal coding (Figure 1D-E). For example, Schneegans and Bays (2017) argued that active and latent items are encoded in the same neural patterns and differ only quantitatively in their level of activation (see also Chun, 2011; Kiyonaga & Egner, 2013). They describe an attractor model with an attention parameter that modulates the gain of activity coding for items cued as relevant, compared to latent items (Figure 1D, left). Importantly, persistent activity of items prior to cue presentation is just attenuated, not abolished. If the latent item becomes relevant, activity is increased to the activation level of an active item, allowing more accurate readout. It is worth noting that this model implies that basic maintenance only requires a relatively low energy persistent activity state, whereas the additional activity for attended/active items serves a distinct purpose (e.g., to allow for more reliable readout of the attended item).

In contrast to the orthogonal coding schemes listed above, a difference in activity level between active and latent items means that the underlying patterns are positively correlated (Figure 1D, middle), which presumably could lead to greater cross-talk between active and latent states (Figure 1D, right). For example, the latent item could distort the readout population's estimate of the active item, or create confusion between active and latent items. The severity of this confusion should depend on the relative activation strength of the latent item. One consequence of this should be a trade-off between confusability with the latent item, and more general durability of the memory. While this trade-off could help explain classic WM capacity limits (Ma et al., 2014), there is some behavioural evidence that formerly latent items can be restored to an active status with little information loss (Hollingworth & Maxcey-Richard, 2013; Rerko & Oberauer, 2013; also Oberauer, 2002; and Nee & Jonides, 2014; but see, e.g., Rerko et al., 2014, for costs to latent WM).

Another non-orthogonal coding scheme for active and latent items is suppressive coding. Suppressive coding has been recognized as a general feature of WM delay activity. This manifests as a reversal of selectivity of a proportion of neurons (or of activation patterns measured with BOLD) during a memory delay, relative to encoding. Reversed selectivity is consistent with the idea that some memory-selective cells reduce their firing rate below baseline during WM maintenance. This effect has been demonstrated in sensory areas (Linke et al., 2011; Lorenc et al., 2020; Zaksas & Pasternak, 2006), prefrontal cortex (Fuster, 1973; Hussar & Pasternak, 2012; Lara & Wallis, 2014; Zhou et al., 2012), and parietal cortex (Zhou et al., 2012). Building on activity reversals relative to stimulus encoding, two recent fMRI studies have suggested that a WM item can also reverse its activity profile between active and latent states (van Loon et al., 2018; Yu et al., 2020). The studies measured BOLD signals in visual and parietal cortex to decode active and latent WM items, and found that when a classifier was trained to discriminate the active WM and was then applied to identify the latent item, its performance dropped below chance level. In other words, item-specific patterns are anti-correlated between the active and latent state. One mechanism to achieve this could be suppression of item-selective neurons when that item enters a latent state (Figure 1E, left and middle).

A suppression or reversal of coding for latent items might seem like a means of reducing their influence on behaviour. However, even a negative relationship means that a meaningful portion of the activity related to the latent item falls into the subspace coding for the active item (Figure 1E, right). This negative correlation potentially suffers from the same problem as any mechanism relying on positively correlated patterns between active and latent states: readout trained to discriminate the active item might be influenced by the identity of the latent item. In particular, suppressive coding of the latent item should drive readout of the active item to be *less* similar to the latent one than it actually was, possibly leading to mnemonic repulsion between active and latent items. Interestingly, such repulsion has been reported in certain cases at the behavioural level (e.g., Myers et al., 2018; see also Almeida et al., 2015; Nassar et al., 2018). This points to the possibility that suppressive coding could be adaptive in some task environments when similar items need to be disambiguated (e.g., Geng et al., 2017). More generally, non-orthogonal coding mechanisms could explain why behaviour can be temporarily guided by latent WM (Mallett & Lewis-Peacock, 2018).

## Temporal separation via oscillations

An alternate form of separating active and latent WM contents is via temporal coding, for instance through periodic reactivation of individual WM items at different phases of an ongoing slow oscillation (Axmacher et al., 2010; Bahramisharif et al., 2018; Jensen & Lisman, 1996; Lisman & Idiart, 1995). To our knowledge, phase separation of active and latent WM items has not yet been demonstrated, but this appears to be a plausible mechanism. For instance, inter-areal phase synchronization (Johnson et al., 2018; Spellman et al., 2015) could ensure that a downstream population is driven only by item reactivations at the optimal phase (reserved for the active item), while latent items reactivate at suboptimal phases, ensuring minimal interference with subsequent processing. This would achieve a similar aim as segregating activity into distinct subspaces.

## Transforming latent into active WM

Our aim is not to advocate for any specific means of distinguishing active from latent WM. However, it is worth considering how plausibly the possibilities outlined above could fulfil one key demand: transferring WM items from a latent to an active state (or vice versa). The separation by neurophysiological mechanism (activity vs activity-silent storage) seems to provide the most straightforward solution: a latent WM item can be moved to an active state simply by activating the population storing the latent item in its synaptic weights. Several computational models have shown that pattern completion can lead a population into an attractor state coding for the now active item (Manohar et al., 2019; Oberauer & Lin, 2017). Attentional gain coding and suppressive coding could also allow for relatively straightforward transformations, by either amplifying existing activity (attentional gain) or rebounding from a suppression of activity. By contrast, anatomical separation requires an apparently more complex transfer of information from one brain area (coding for latent WM) to another (coding for active WM). While this has not been demonstrated for WM, it has been shown in other contexts (e.g., Crowe et al., 2013). The study by Christophel et al. (2018) also strongly suggests this possibility, since their paradigm required maintaining two items until a retrospective cue indicated which to prioritize, presumably triggering its reactivation in visual cortex. Similarly, separation by activity subspace within a brain area faces the challenge of shifting neural activity from one subspace to another. While this has not been demonstrated specifically for transitions of latent to active WM, it has been observed in a number of other contexts (e.g., Tang et al., 2019), and particular in transitioning representations from a neutral to a prioritized state after a retrospective cue (Panichello & Buschman, 2020). Since the mechanism for moving information across brain areas or subspaces is unknown, it cannot be ruled out that such a transformation could reintroduce interference between active and latent WM that had been avoided by orthogonalising them in the first place.

## Future directions

We have laid out a variety of theoretical mechanisms for the storage of functionally active or latent items in WM. Since the key constraint for latent items is that they should not interfere with current behaviour or storage of the active item, the main constraint on possible storage mechanisms is not whether it is persistently active or activity-silent, but rather the orthogonality of the respective coding schemes. Although separation by neurophysiological mechanisms (persistent activity vs activity-silent maintenance) could fulfil this key constraint, it is only one of a larger set of possible solutions. Importantly, it is possible that active and latent items could both be maintained using the same kind of activity state (persistent activity or activity silent maintenance), so long as the mnemonic states are orthogonal: e.g., separate brain areas, overlapping but distinct neural populations, or non-overlapping activity subspaces of the same population.

The distinction between active and latent items echoes the distinction between attentional templates and accessory memory items made by Olivers et al. (2011). The authors distinguished between different neural mechanisms that could underlie the storage of accessory memory items so that it does not drive attentional capture. Our framework can be thought of as an extension of this idea. Attentional capture by stimuli matching the active item (but not the latent item) is one means by which the active item may influence cognition or behaviour. In this instance, the maintenance of the active item would be expected to exert top-down influence on sensory areas so that they preferentially process matching input. In a more general framework, this can be interpreted as one of several possible downstream consequences of the active item that needs to be avoided by the latent item. As we showed recently (Muhle-Karbe et al., 2020), the same principle should also extend to WM-based decision-making (see also Myers et al., 2015).

Identifying which of the outlined mechanisms support the distinction between active and latent WM requires robust methods for the identification of neural coding mechanisms. However, significant challenges remain for testing candidate neurophysiological coding schemes such as persistent activity and activity-silent coding. In particular, activity-silent states are fundamentally challenging to infer, given that most methods in neuroscience measure some form (or correlate) of neural activity. Recently, we developed an impulse-response approach to “ping” activity-silent neural states by measuring the brain's response to task-irrelevant driving input, providing a theoretical potential to track the behaviour of a greater variety of mnemonic states (Wolff et al., 2015; Wolff et al., 2017; see also Rose et al., 2016). While this approach will be useful for enhancing our sensitivity for detecting memory signals that are otherwise undetectable (for whatever reason), on its own it does not strictly adjudicate between the alternative neurophysiological mechanisms. For instance, Schneegans and Bays (2017) have illustrated how low levels of persistent activity could underlie an apparently activity-silent code. Nevertheless, this approach can provide a useful complement to more standard measures of delay-period activity because it more sensitively detects activity related to latent WM items (Wolff et al., 2017). This, in turn, could be used to test more in-depth questions, such as whether active and latent items share the same code.

Definitive evidence for activity-silent mechanisms will ultimately require specific evidence of the supposed underlying processes, such as temporary connectivity changes (e.g., periodic refreshing or reactivation of a memory representation, Mongillo et al., 2008), or intrinsic gain modulation (e.g., Stroud et al., 2018). Moreover, the ability to identify how the brain separates WM representations will always be limited by what current methods can detect. Similarly, even when items appear to share the same coding mechanism, we cannot rule out undiscovered differences in coding that are invisible to current methods. At the same time, inferring persistent, uninterrupted activity is not trivial either. Elevated firing during delay periods could reflect transient non-maintenance processes, which can appear to be persistent firing when averaged over many trials (Miller et al., 2018).

More generally, it may be insufficient to rely on decodability alone to infer a putative mnemonic state. Decodability has become a ubiquitous marker of WM maintenance (for an overview on WM and decoding, see Christophel et al., 2017), but we propose that future work will need to focus less on the mere presence or absence of decodable neural patterns, and more on the functional properties of candidate neural states. This is important because a decodable neural state could be epiphenomenal to WM (e.g., reflect mental imagery or probe expectation, rather than maintenance per se, see Stokes, 2011). Moreover, even if the neural state is necessary for WM, it is still critical to understand *how* it influences neural response dynamics to gain a mechanistic understanding of the underlying process (rather than simply identifying the brain area maintaining the WM engram).

## Conclusions

In conclusion, we caution against a direct equivalence between functional states in working memory and their corresponding neural states. The key theoretical constraint is that active and latent WMs should be maintained via qualitatively distinct neural states. Within these theoretical constraints, the precise mechanisms of maintenance for either type of WM remains an empirical question. There remain major challenges associated with establishing the neurophysiological mechanisms of maintenance. We argue that focusing on the functional behaviour of putative mnemonic states will be an important future direction.

## Note

1 Note that related distinctions between priority states in WM have previously been made. Classic WM models distinguish between attended and unattended items (Cowan, 2001; Oberauer, 2002), between items that guide attention and those that do not (Olivers et al., 2011), or between prioritized and (temporarily) unprioritized items (Lewis-Peacock et al., 2012). In the present article, we focus on the functional differences between representational states, i.e., how they differentially affect subsequent processing (see also Myers et al., 2017). Therefore, here we prefer to use the terms “active” and “latent” to emphasize functional consequences and to avoid connoting a particular role for attention in the process.
